# The cytoplasmic poly(A) polymerases GLD-2 and GLD-4 promote general gene expression via distinct mechanisms

**DOI:** 10.1093/nar/gku838

**Published:** 2014-09-12

**Authors:** Marco Nousch, Assa Yeroslaviz, Bianca Habermann, Christian R. Eckmann

**Affiliations:** 1 Max Planck Institute of Molecular Cell Biology and Genetics (MPI-CBG), Pfotenhauerstrasse 108, Dresden, 01307, Germany; 2 Max Planck Institute of Biochemistry (MPIB), Am Klopferspitz 18, Martinsried, 82152, Germany

## Abstract

Post-transcriptional gene regulation mechanisms decide on cellular mRNA activities. Essential gatekeepers of post-transcriptional mRNA regulation are broadly conserved mRNA-modifying enzymes, such as cytoplasmic poly(A) polymerases (cytoPAPs). Although these non-canonical nucleotidyltransferases efficiently elongate mRNA poly(A) tails in artificial tethering assays, we still know little about their global impact on poly(A) metabolism and their individual molecular roles in promoting protein production in organisms. Here, we use the animal model *Caenorhabditis elegans* to investigate the global mechanisms of two germline-enriched cytoPAPs, GLD-2 and GLD-4, by combining polysome profiling with RNA sequencing. Our analyses suggest that GLD-2 activity mediates mRNA stability of many translationally repressed mRNAs. This correlates with a general shortening of long poly(A) tails in *gld-2*-compromised animals, suggesting that most if not all targets are stabilized via robust GLD-2-mediated polyadenylation. By contrast, only mild polyadenylation defects are found in *gld-4*-compromised animals and few mRNAs change in abundance. Interestingly, we detect a reduced number of polysomes in *gld-4* mutants and GLD-4 protein co-sediments with polysomes, which together suggest that GLD-4 might stimulate or maintain translation directly. Our combined data show that distinct cytoPAPs employ different RNA-regulatory mechanisms to promote gene expression, offering new insights into translational activation of mRNAs.

## INTRODUCTION

One of the unifying features of living organisms is their ability to regulate gene expression programs. Prior to protein production, many regulatory possibilities exist that operate at the transcriptional or post-transcriptional level, which are mediated by mechanisms that target DNA or RNA, respectively. The global importance of these two distinct regulatory modes appears to differ between tissues. Many somatic cell types primarily use transcriptional control mechanisms, whereas numerous examples in neurons and germ cells highlight a prevalence of post-transcriptional control mechanisms. However, the underlying functional mechanisms and their contribution to the global level of post-transcriptional gene expression regulation remain to be determined.

Messenger RNAs (mRNAs) mature in the nucleus and serve as templates for ribosome-mediated protein synthesis in the cytoplasm. However, mRNAs subjected to post-transcriptional control are withheld from entering the translational pool via targeted degradation or translational repression. Although both processes result in the down-regulation of protein levels, they are mechanistically distinct: mRNA degradation results in the final destruction of the mRNA template and is irreversible; translational repression stabilizes the target and is reversed in a process termed translational activation. Due to the flexibility of translational control, mRNA repression and activation mechanisms provide an immediate mode of gene expression regulation in dynamic biological systems.

An mRNA intrinsic feature that registers repressive and active translational control mechanisms is its poly(A) tail. Originally added in the nucleus (1), this homopolymer of adenosines at the 3′end is subject to distinct length changes influencing cytoplasmic mRNA fates. Especially in animals, poly(A) tail shortening is a key step in the mRNA decay pathway (2), establishing a clear relationship between longer poly(A) tail lengths and increased stability. Poly(A) tail length is also correlated with translational efficiency. Longer poly(A) tails enhance protein synthesis in many *in vitro* translation extracts (3). Furthermore, in developmental contexts gene expression regulation is strongly connected to poly(A) tail extension. For example, during early *Drosophila* embryogenesis or *Xenopus* oocyte maturation, gene-specific poly(A) shortening or elongation correlates with the protein amounts needed in the following developmental stage (4,5). This leads to the generalization that mRNAs with long poly(A) tails are better translated than short ones. However, no global correlation between poly(A) tail length and translation efficiency is observed in somatic tissue culture systems or during late stages of embryonic development (6). Only during early developmental stages, when transcriptional regulation is not present yet, a strong correlation between long poly(A) tails and high translational efficiency is detected (6). This suggests that global mechanisms of gene expression regulation, involving mRNA poly(A) tail-length changes, are best revealed in systems where post-transcriptional control is the dominant mode of gene expression.

**Figure 1.**
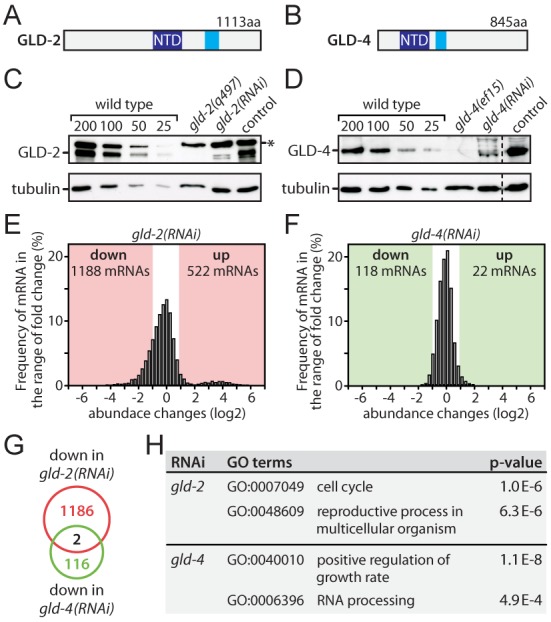
*gld-2* but not *gld-4* promotes mRNA abundance. (**A** and **B**) Domain structure of GLD-2 and GLD-4 proteins: dark blue–nucleotidyl transferase domain (NTD), light blue–poly(A) polymerase-associated domain. (**C** and **D**) Western blot analysis of protein levels in RNAi-treated adults. The asterisk marks a non-specific background band. Representative images are shown (n = 3). (**E** and **F**) mRNA abundance changes in RNAi-treated animals. All detectable 7649 genes are shown and the number of significant abundance changes is indicated. (**G**) The overlap of down-regulated genes between *gld-2(RNAi)* and *gld-4(RNAi)* is shown. (**H**) A GO-term analysis for *gld-2(RNAi)* and *gld-4(RNAi)* down-regulated genes was conducted and two representative categories are shown for each.

Cytoplasmic poly(A) polymerases (cytoPAPs) represent a class of enzymes that are proposed to post-transcriptionally elongated poly(A) tails of mRNAs. Two cytoPAPs have been described so far in animals, GLD-2 and GLD-4 (7–9). Both proteins belong to two distinct, evolutionary conserved protein families of non-canonical nucleotidyltransferases that contain no sequence homology outside their enzymatic regions (10) (Figure 1A and B). Moreover, both lack predictable RNA-binding domains and, therefore, are hypothesized to rely on interactions with RNA-binding proteins to establish efficient contact with mRNA targets (7,8). For GLD-2, strong polyadenylation activity has been detected in tethering assays when probing the nematode, fly, frog and mammalian homologs (11,12). For GLD-4, only the enzyme of the nematode *Caenorhabditis elegans* was tested in such an assay; polyadenylation depended on its intact nucleotidytransferase domain and required the species-specific co-factor, GLS-1 (8). The *in vivo* function of either GLD-2 or GLD-4 as cytoplasmic poly(A) polymerases is apparent from the polyadenylation defects of specific mRNA targets in the corresponding mutants or RNAi-mediated knockdowns (9,13–19). Although it was recently suggested that GLD-2 might target numerous mRNAs in *C. elegans* and *Drosophila melanogaster* (14,18), very few mRNA targets have been reported for *C. elegans* and human GLD-4 (8,9,15,19). The global role(s) and functional mechanism(s) of either cytoPAP still remain(s) unclear.

In this work, we address the role of *C. elegans* GLD-2 and GLD-4 in global mRNA regulation. We combine polysome profiling with RNA sequencing to identify GLD-2- and GLD-4-dependent changes in mRNA abundance and translation. We find that GLD-2 primarily stabilizes mRNAs that are translationally repressed. Furthermore, GLD-2 strongly promotes bulk polyadenylation. Surprisingly, these functions of GLD-2 seem to have little impact on stimulating efficient target mRNA translation. By contrast, we find that GLD-4 promotes bulk polyadenylation only mildly and has no major role in promoting general mRNA stability. However, GLD-4 is needed for efficient polysome formation and general mRNA translation. Taken together, our data indicate that GLD-2 and GLD-4 use two distinct mechanisms to promote gene expression in germ cells.

## MATERIALS AND METHODS

### Strains and RNAi feeding

Worms were handled according to standard procedures and grown at 20°C unless otherwise stated (20). Mutations used in this study: LG *I*: *gld-2(q497)*, *gld-4(ef15)*, *gls-1(ef8)*, LG *III*: *glp-1(q224ts)*. With the exception of the *glp-1* mutation, all others were kept as heterozygotes over the *hT2[qIs48] I;III* balancer and homozygote F1 progeny was analyzed. Bristol N2 served as the reference wild-type strain. For all analysis, we synchronized L1 animals by starvation and harvested them after growing on feeding plates 24 h after the mid-L4 stage. The feeding constructs against *ccr-4*, *ccf-1*, *gld-4* and *fem-3* were described previously (8,21,22). The empty pL4440 vector represented control RNAi. The plasmids were transformed into HT115(DE) *Escherichia coli* cells and double-stranded RNA production was induced with IPTG according to standard methods.

### Immunoblotting and antibodies

Primary antibodies against the following proteins were used: anti-GLD-2 (19), anti-GLD-4 (8), anti-α-tubulin (T5168, Sigma), rabbit anti-GLS-1 (22), anti-eIF2α (23) and anti-GST (MPI-CBG, antibody facility). Polyclonal antibodies against the two similar proteins PAB-1 and PAB-2 were generated by immunizing rabbits with a recombinant GST-tagged fusion peptide of PAB-2 (aa 517 to 692). The serum's specificity and cross-reactivity was verified by comparing *pab-1(RNAi)* or *pab-2(ok1851)* mutant extracts with that of wild-type. Horseradish peroxidase-conjugated secondary antibodies were purchased from Jackson ImmunoResearch.

### Sucrose gradient centrifugation

Whole worm extracts were prepared from age-matched adult worms as previously described (24). Equal amounts of total cellular protein (20 mg) were resolved through a 10 ml 17–50% sucrose gradient (25). The gradients were spun for 210 min at 30 000 rpm and 4°C in a SW40Ti rotor (Beckman Coulter). The fractionation was conducted bottom up while the absorbance profile at 260 nm was recorded. For RNA isolations, the samples were fractionated directly into three volumes of ethanol, and 150 pg of *in vitro* transcribed firefly luciferase mRNA (FFluc) was added to each fraction. No FFluc spike-in mRNA was added to the input samples. For protein analysis, 50 ng of purified GST peptide was added to each fraction as a proxy to survey sample precipitation efficiency. GST-protein purification was conducted as previously described (25).

### RNA handling and bulk poly(A) tail length measurements

The ethanol/gradient mixture of input and gradient material was incubated overnight at −20°C and pelleted in a benchtop centrifuge for 20 min at 16 000 x *g* and 4°C. The pellets were TRIzol (Invitrogen) extracted following the manufacturer's protocols. The resulting RNA was either sequenced by the Dresden Genome Center on an HiSeq2000 (Illumina) platform or reverse transcribed using random hexamer primers and RevertAid Premium reverse transcriptase (Fermentas), according to the manufacturer's protocols. Quantitative PCR (qPCR) was conducted on a Mx3000P qPCR system (Stratagene) using the ABsolute QPCR SYBR Green mix (Thermo) and gene-specific primers (sequences available upon request). All gradient data were normalized to the FFluc spike-in mRNA control. For bulk poly(A) tail measurements, total RNA was isolated from hand-picked adults using the TRIzol method. One microgram of total RNA was used in the 3′ end labeling assay and processed according to the published methods (26), with the only exception that un-incorporated α^32^P-Cordycepin (Perkin Elmer) was removed using Mini Quick Spin Columns (Roche).

### Library preparation and next-generation sequencing (NGS)

mRNA was isolated from 2 ug of total RNA by poly-dT25 enrichment using the NEBNext Poly(A) mRNA Magnetic Isolation Module according to the manufacturer's instructions and eluted in 15 ul 2-times first-strand cDNA synthesis buffer (NEBNext, NEB). After chemical fragmentation for 15 min at 94°C, the sample was directly subjected to the workflow for strand-specific RNA-Seq library preparation (Ultra Directional RNA Library Prep, NEB). After ligation of custom adaptors, unused adapters were depleted by a 1-times XP-bead purification (Beckman Coulter); adaptor-oligo1: 5′-ACACTCTTTCCCTACACGACGCTCTTCCGATCT-3′, adaptor-oligo2: 5′-P-GATCGGAAGAGCACACGTCTGAACTCCAGTCAC-3′. Indexing was done during the following PCR enrichment (15 cycles) using custom amplification primers, carrying the index sequence indicated with ‘NNNNNN’; primer1: AATGATACGGCGACCACCGAGATCTACACTCTTTCCCTACACGACGCTCTTCCGATCT, primer2: GTGACTGGAGTTCAGACGTGTGCTCTTCCGATCT, primer3: CAAGCAGAAGACGGCATACGAGATNNNNNNGTGACTGGAGTT. After two more 1-times XP-bead purifications, libraries were quantified using the Qubit dsDNA HS Assay Kit (Invitrogen). For Illumina flowcell production, samples were equimolarly pooled and distributed on all lanes used for 75-bp single-read sequencing on Illumina HiSeq 2000.

### Analysis of NGS data

The quality of the NGS data was analyzed using the fastqc software (http://www.bioinformatics.babraham.ac.uk/projects/fastqc/). TruSeq adapter were removed using cutadapt (version 1.2.1) (27). The fastq files were mapped to the *C. elegans* genome downloaded from Ensembl (WS220) using the TopHat2 algorithm (version v2.0.10) allowing only unique mapping (28). Using the featureCount algorithm from the SubRead software package (version 1.4.0), the reads were counted from the bam files (tophat2 output) on exon-level based on the gene annotation from Ensembl, resulting in a read count for each gene (29). Using the DESeq package (R 3.0.2, DESeq version 1_14_0), the count data were normalized by the size factor to estimate the effective library size (30). After calculating the gene dispersion across all samples, the comparison of two different conditions resulted in a list of differentially expressed genes. Genes with a normalized read count smaller than 100 were ignored in the final analysis. A pre-filtering step was used to calculate the number of genes showing a high probability of being differentially regulated. In this step, genes with a probability (unadjusted *P*-value) above 0.003 were excluded from the differential expression analysis. Genes with a fold-change higher or equal to two, as well as an adjusted *P*-value of < = 0.05 was then defined as differentially expressed. The *P*-values are being adjusted to the multiple testing hypotheses to reduce the false discovery rate (FDR). For the analysis of the translation efficiency, we needed to correct the analysis for the bias between the non-polysomal (NP) and polysomal (P) fractions. We calculated a correction factor based on the amounts of FFluc spike-in to adjust the NP and P data for each sample.

## RESULTS

### GLD-2, but not GLD-4 activity stabilizes mRNAs

The animal model, *C. elegans*, is a well-established organism to study post-transcriptional gene regulation in germ cells (31). The adult animal is a self-fertile hermaphrodite and its fully developed germline tissue constitutes a large proportion of its biomass, allowing for the detection of germline-specific gene expression changes even in whole animal extracts. GLD-2 and GLD-4 are predominantly but not exclusively expressed in the germ line, and are present at almost all stages during germ cell development (7,8). To assess and compare the influence of the two cytoplasmic poly(A) polymerases on general mRNA abundance, we performed RNAi-knockdown experiments followed by RNA sequencing. The technique of RNAi feeding was used because large quantities of synchronized animals were needed for our experiments, which is difficult to obtain from *gld-2* and *gld-4* homozygote mutants.

We analyzed young adults that were fed with either control RNAi, *gld-2(RNAi)* or *gld-4(RNAi)*. A strong cytoPAP protein knockdown was observed by western blot analysis: less than ∼10% of both GLD-2 (Figure 1C and Supplementary Figure S1A) and GLD-4 (Figure 1D and Supplementary Figure S1A) remained in RNAi-treated animals. In agreement with previous studies, *gld-2(RNAi)*-treated animals were sterile with somewhat less severe germline phenotypes compared to the genetic null mutant *gld-2(q497)* (data not shown) (14). The milder *gld-2(RNAi)* phenotype is considered an advantage for the expression analysis as it presumably reduces indirect effects arising from developmental changes during late oogenesis. In comparison to the strong-loss-of-function mutant *gld-4(ef15)* and consistent with previous observations (8,19), the germline defects of *gld-4(RNAi)* animals were less severe and the animals were fertile. No overt somatic defects were apparent in either cytoPAP RNAi-treated animals.

Next, we measured mRNA levels by isolating total RNA from RNAi-treated animal extracts, followed by mRNA enrichment and non-strand-specific RNA sequencing. In *gld-2(RNAi)*, reads could be mapped to 15 620 genes. In *gld-4(RNAi)*, 11 791 genes could be detected (Supplementary Data S1). Both gene lists were combined, and after removal of low expressing genes, 7649 high confidence genes were used for further analysis (Supplementary Data S1). When comparing *gld-2(RNAi)* to control RNAi, widespread RNA changes in abundance were observed: 1188 mRNAs were reduced and 522 mRNAs increased upon GLD-2 reduction (Figure 1E). By contrast, overall mRNA abundance changes in *gld-4(RNAi)* were much less frequent and less strong in amplitude: 118 mRNAs were reduced and 22 mRNA are increased (Figure 1F and Supplementary Figure S1B). The overlaps between the *gld-2(RNAi)* and *gld-4(RNAi)* expression changes were quite small (Figure 1G and Supplementary Figure S1C–E), suggesting that distinct mRNA populations are affected by the reduction of either cytoPAP protein. This view is further supported by a Gene Ontology (GO) annotation analysis of down-regulated mRNAs in *gld-2(RNAi)* and *gld-4(RNAi)* showing that different biological processes are affected (Figure 1H). A GO-term analysis of the *gld-2(RNAi)* up-regulated genes yielded as the top-scoring term pseudopodium (GO:0031143) (data not shown), pseudopod formation is the final process of spermatogenesis, suggesting that many of these genes might be increased due to indirect effects.

**Figure 2.**
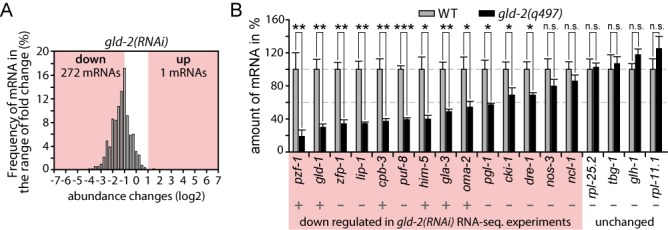
GLD-2-associated mRNAs are less abundant. (**A**) Abundance changes of 538 previously described GLD-2-associated mRNAs (14) in our *gld-2(RNAi)* dataset. (**B**) RT-qPCR measurements of randomly selected down-regulated and unchanged genes, comparing mRNA abundance in *gld-2(q497)* mutants to wild-type. A plus sign indicates previously proposed GLD-2-associated mRNAs (14). Shown is the mean (±SD) of three independent experiments. Significance was calculated with a Student's *t*-test: ***, *P* < 0.001; **, *P* < 0.01; *, *P* < 0.05; n.s., not significant.

To focus our analysis on mRNA expression changes in the germ line, we conducted the same analysis on 2243 previously defined germline-enriched genes (32) (Supplementary Data S1). Similar to the entire dataset, germline genes show broad expression changes in *gld-2(RNAi)* with 444 mRNAs down and 283 up-regulated (Supplementary Figure S1F), and only mild changes in *gld-4(RNAi)* with 54 mRNA down and 7 up-regulated (Supplementary Figure S1G). We find that the majority of up-regulated mRNAs in the *gld-2(RNAi)* are functionally connected to spermatogenesis (237 out of 283 genes). This is consistent with *gld-2* null mutant defects (33), and the observed sterility in *gld-2(RNAi)* animals, which is partly due to spermatogenesis arrested germ cells. By contrast, wild-type germ cells complete spermatogenesis in the last larval stage before adulthood to produce fertilization-competent spermatozoa. Therefore, the increase of spermatogenic mRNAs in *gld-2(RNAi)* animals is likely due to later developmental arrests that are secondary to an initial molecular requirement of GLD-2 during earlier stages of spermatogenesis. Alternatively, indirect effects may also arise from GLD-2's function to promote a negative regulator of RNA stability, as documented for hGLD2 (9). Moreover, some down-regulated mRNAs are likely indirect due to changes in female germline development. However, we also noticed that *gld-2*-dependent mRNAs are enriched for previously identified GLD-2-associated mRNAs; of the 538 GLD-2-associated mRNAs, 272 are significantly reduced in our complete dataset (Figure 2A and Supplementary Figure S1H) (14), arguing that they likely represent direct targets of GLD-2 control. Hence, throughout the rest of this work, we will refer to them as GLD-2-stabilized mRNAs. To test whether the decrease in mRNA abundance is due to a bias in the RNA sequencing procedure, we analyzed the mRNA levels of 18 exemplary genes via quantitative RT-PCR (qRT-PCR), using random hexamers for reverse transcription. Most down-regulated mRNAs (12/14) and none of the negative controls (4/4) were significantly reduced in *gld-2*-compromised animals (Figure 2B). Taken together, these data suggest that *gld-2* but not *gld-4* activity is important to maintain or up-regulate the abundance of many mRNAs.

### GLD-2 but not GLD-4 promotes strong bulk poly(A) tail elongation

The poly(A) tail status of several developmentally and post-transcriptionally controlled germ cell mRNAs depends on *gld-2* and *gld-4* activity (13–15). To assess whether these findings are also detectable on a global scale, we performed bulk poly(A) tail measurements. To this end, total RNA was isolated from different genotypes or RNAi-treated young adults, radioactively end-labeled with cordycepin, partially digested with RNases A/T1 and the remaining poly(A) sequences were analyzed on sequencing gels (21). Due to the sensitivity of this method, we also included genetic mutations of *gld-2* and *gld-4* to strengthen our analysis.

**Figure 3.**
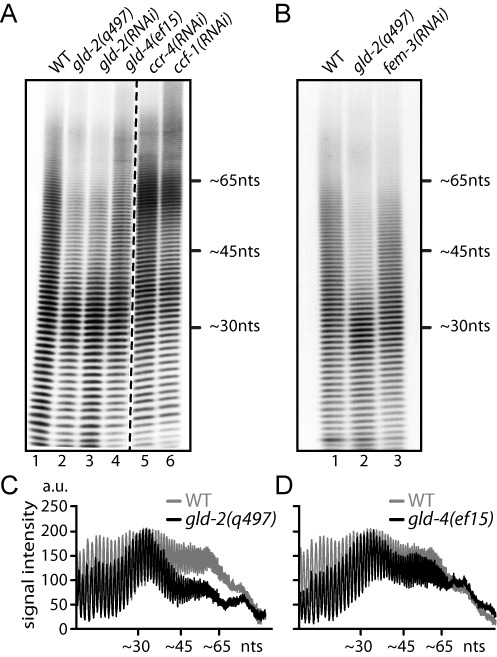
*gld-2* promotes bulk mRNA poly(A) tail extension. (**A** and **B**) Representative gels of at least three independent bulk poly(A) tail measurements. Equal amounts of radioactivity were loaded for each sample. WT, wild-type. (**C** and **D**) Line scans of bulk poly(A) profiles from (A).

In the wild-type, bulk poly(A) tails extended on average from ∼20 up to ∼100nt in length (Figure 3A and B, lane 1). As previously described (21), this distribution is shifted toward longer tails in deadenylation-compromised *ccr-4(RNAi)* or *ccf-1(RNAi)* animals (Figure 3A, lane 5 and 6). The opposite effect was observed in *gld-2(q497)* mutants and *gld-2(RNAi)* animals: bulk poly(A) tails were shortened to ∼20–40 nucleotides (nts), and poly(A) tails above ∼40 nts were strongly reduced in abundance (Figure 3A, lane 2 and 3, and 3C). An obvious difference between *gld-2*-compromised animals to wild-type is the absence of embryos (33). To exclude that a lack of embryos may have biased our measurements, we analyzed *fem-3(RNAi)* animals that produce no embryos, due to the lack of sperm (34). However, the overall poly(A) tail profile of feminized animals was comparable to wild-type (Figure 3B, compare lanes 1 and 3, and Supplementary Figure S2C), suggesting that the observed dramatic *gld-2*-dependent poly(A) tail shortening is not a consequence of sterility.

In *gld-4(ef15)* mutants and *gld-4(RNAi)* animals, only a mild overall reduction of bulk poly(A) tails was detected (Figure 3A, lane 4, and 3D; data not shown). In comparison to wild-type, the overall *gld-4* poly(A) profile primarily differed in a reduction of lengths below ∼35 nts. Compared to *gld-2*, poly(A) tails beyond ∼40 nts were less severely affected. Last, as GLD-2 and GLD-4 are mainly expressed in germ cells, we assessed the contribution of the germline tissue to bulk poly(A) profiles by comparing wild-type to *glp-1(q224ts)* animals, which at the restrictive temperature do not develop a germ line (35). In germline-less *glp-1(q224ts)* young adults, only a mild reduction of bulk tails was observed compared to wild-type at the non-permissive temperature (Supplementary Figure S2A and B), affirming that our assay in general detects poly(A) changes that primarily originate from the germline tissue. Taken together, our bulk poly(A) measurements reveal that GLD-2 strongly and GLD-4 mildly promote general mRNA polyadenylation in germ cells.

### GLD-2 stabilizes poorly translated mRNAs

**Figure 4.**
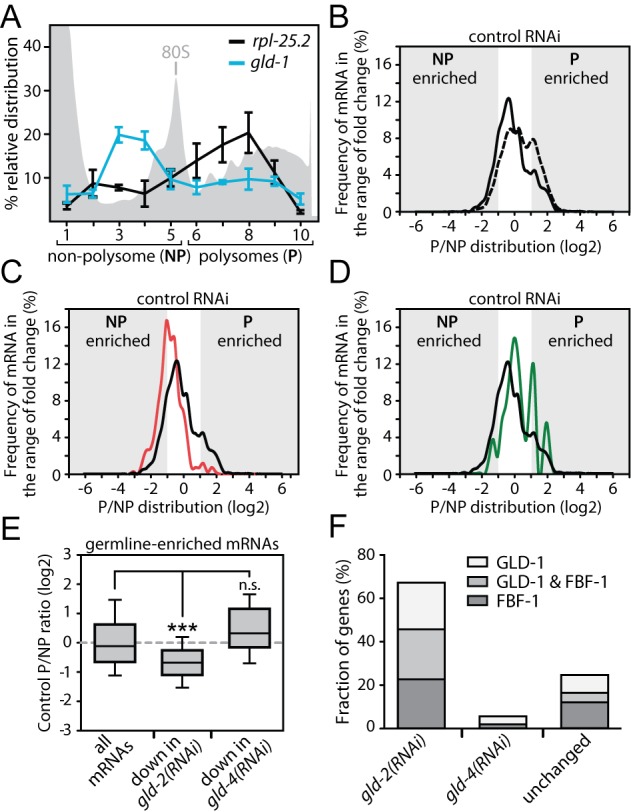
*gld-2* promotes abundance of poorly translated germline mRNAs. (**A**) The relative gradient distribution of *rpl-25.2* and *gld-1* mRNA in animals treated with control RNAi is analyzed by RT-qPCR. Shown is the mean (±SEM) from 10 fractions in three independent experiments. (**B**–**D**) The gradient distribution of different groups of mRNAs was analyzed between polysome (*P*) and non-polysome (*NP*) fractions for control RNAi-treated animals. Shown are (**B**) all detected genes (dotted line–7649 mRNAs), (B, C and D) germline-enriched genes (solid black line–2243 mRNAs), (**C**) germline genes that are less abundant in *gld-2(RNAi)* (solid red line–444 mRNAs) and (**D**) germline genes that are less abundant in *gld-4(RNAi)* (solid green line–54 mRNAs). (**E**) P/NP distribution of the indicated groups of germline mRNAs. Significance was calculated with a Student's *t*-test: ***P < 0.001; n.s., not significant. (**F**) A large percentage of GLD-2-stabilized germline mRNAs are also GLD-1 and FBF-1 targets. The percent overlap between GLD-2-regulated mRNAs and putative GLD-1 and FBF-1 target mRNAs are given. mRNAs that are not decreased in *gld-2(RNAi)* are labeled as unchanged.

An important aspect of translational control is the regulated access of mRNAs to ribosomes. The translational status of mRNAs can be analyzed by polysome profiling. This technique measures the distribution of mRNAs in a sucrose gradient where ribosomes are separated into active and inactive fractions, based on their density and shape (36). We performed this analysis with extracts prepared from synchronized young adults and compared the known translationally regulated *gld-1* mRNA to the presumably unregulated *rpl-25.2* mRNA. The majority of *gld-1* was present in the non-polysomal region, whereas *rpl-25.2* was mainly detected in the polysomal region of the gradient (Figure 4A). This demonstrates that mRNAs subjected to translational control can be clearly separated from unregulated mRNAs.

To analyze the translational status of mRNAs at the global level, we performed RNA-sequencing analysis of mRNAs from pooled fractions of the gradient that correspond to non-polysomal (NP) and polysomal (P) regions (Figure 4A) and mapped 15 849 genes in the control RNAi sample. The relative number of NP- to P-enriched genes is indicative of how many mRNAs are translated with low or high efficiency. In control RNAi, a comparison of the NP- to the P-dataset for all as detectable defined 7649 mRNAs shows that 537 mRNAs are at least 2-fold enriched in the NP-fraction, and 2406 mRNAs in the P-fraction giving an NP/P ratio of 1:4.5 (Figure 4B and Supplementary Data S1), suggesting that the majority of all mRNAs are well translated. A different distribution is observed for germline-enriched mRNAs: 289 versus 404 mRNAs are enriched in the NP- and P-fraction, respectively, resulting in an NP/P ratio of 1:1.4 (Figure 4B and E). This indicates a shift toward non-polysomal enrichments for germline mRNAs. This trend is even more distinct for GLD-2-stabilized germline mRNAs: 133 versus 13 genes are enriched in the NP- and P-fraction, respectively, resulting in an NP/P ratio of ∼10:1 (Figure 4C and E). By contrast, GLD-4-stabilized germline genes do not show this behavior: six versus six genes are enriched in the NP- and P-fraction, respectively. With an NP/P ratio of 1:1, they follow the trend of all germline genes (Figure 4D and E). This suggests that germline mRNAs are poorly translated in general. Moreover, only GLD-2 but not GLD-4 preferentially stabilizes germline mRNAs that are translated with low efficiency.

mRNAs that accumulate in the NP fraction might be targets of translational repression. Hence, we compared the overlap of less abundant germline mRNAs in *gld-2(RNAi)* and *gld-4(RNAi)* to published whole-genome interaction studies of two well-characterized translational repressors in *C. elegans* germ cells, GLD-1 and FBF-1 (37–39). We found that a large number of GLD-2-stabilized mRNAs are also putative GLD-1 and FBF-1 targets (Figure 4F). This is not the case for GLD-4-stabilized or *gld-2*-insensitive mRNAs (Figure 4F), suggesting that GLD-2 but not GLD-4 cytoPAP preferentially sustains the levels of translationally repressed mRNAs.

### GLD-4 promotes general translation efficiency of mRNAs

**Figure 5.**
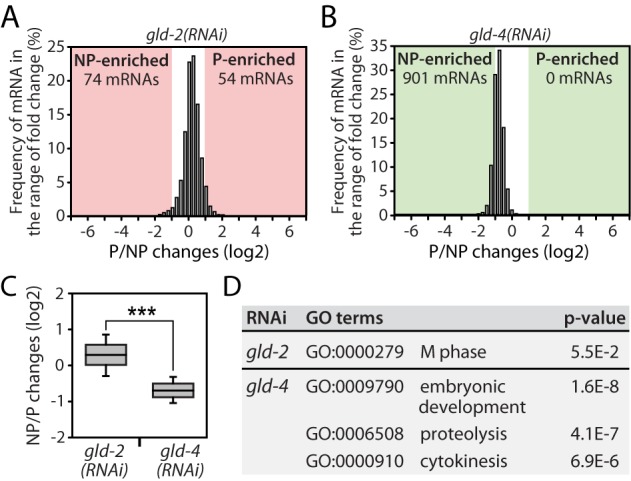
*gld-4* promotes general translation. (**A** and**B**) Translational efficiency changes of all 7649 detectable genes in (A) *gld-2(RNAi)* and (B) *gld-4(RNAi).* (**C**) Statistical analysis of translational efficiency changes. (**D**) GO-term analysis of NP-enriched mRNAs in *gld-2(RNAi)* and *gld-4(RNAi)*. Student's *t*-test: ***, *P* < 0.001.

The poly(A) tail serves to stabilize mRNAs and is implicated in the efficient recruitment of ribosomes (3). To test whether any of the two cytoPAPs has a role in promoting translation, we conducted a gradient analysis of *gld-2(RNAi)* and *gld-4(RNAi)* mRNAs and compared it to control RNAi datasets. We could map reads for 15 907 genes in the *gld-2(RNAi)* sample and 15 899 genes in the *gld-4(RNAi)* sample. We started the analysis with the 7649 genes that were classified as expressed in the input analysis. In *gld-2(RNAi)*, only minor translational changes could be detected: 74 mRNAs were increased in the NP-fraction, and 54 mRNAs in the P-fraction (Figure 5A and C). Interestingly, in *gld-4(RNAi)* the overall translation status of mRNAs is shifted toward lighter fractions with 901 mRNAs being significantly enriched in the NP-fraction (Figure 5B and C), suggesting that many mRNAs are less well translated. Similar trends were observed in *gld-2(RNAi)* and *gld-4(RNAi)* for the 2243 defined germline-enriched genes (data not shown). A GO-term analysis of less well-translated mRNAs suggests that different groups of mRNAs are affected in *gld-2*- and *gld-4*-compromised animals (Figure 5D). Taken together, this analysis argues for a potentially broader role of GLD-4 in promoting mRNA translation than for GLD-2.

To investigate whether mRNAs might rely on a potential combined activity of both cytoPAPs, we ask how many mRNAs are less abundant in *gld-2(RNAi)* and at the same time less well translated in *gld-4(RNAi)*. We found a moderate overlap of 137 mRNAs between these two datasets (Supplementary Figure S3A). A GO-term analysis of these genes revealed an enrichment of genes connected to chromatin organization, the cell cycle and embryonic development (Supplementary Figure S3B), suggesting that a small subset of mRNAs might rely on the combined activity of GLD-2 and GLD-4 for efficient protein expression.

### GLD-4 promotes polysome formation

**Figure 6.**
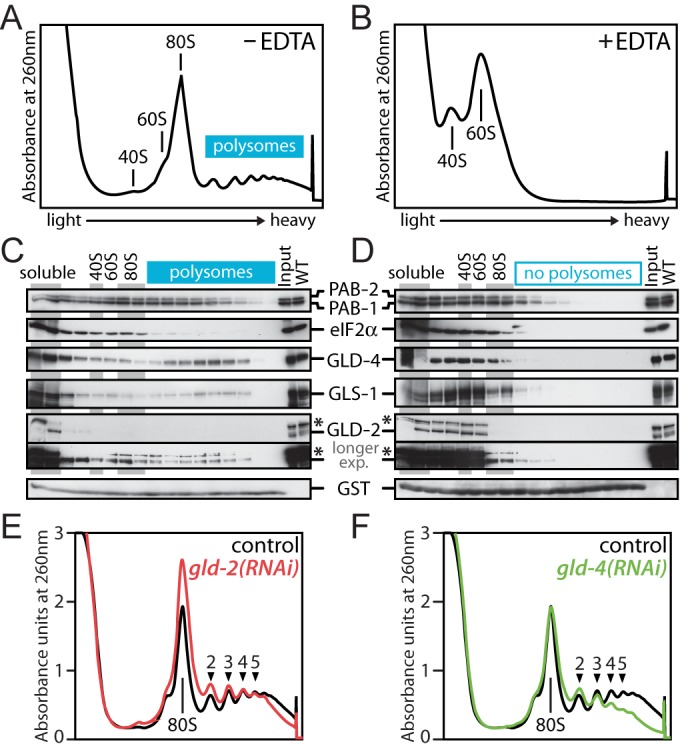
GLD-4 co-sediments with polysomes and promotes polysome formation. (**A** and **B**) Typical absorbance profiles of a wild-type polysome gradient (A) without or (B) with prior EDTA treatment (n = 3). The positions of major ribonucleoprotein complexes are indicated. (**C** and **D**) Western blot analysis of fractionated material from (A) and (B). Equal exposure times for each antibody in (C) and (D); for GLD-2, also a longer exposure of the same blot is shown; asterisk marks an unspecific background band. (**E** and **F**) Representative absorbance profiles from extracts of RNAi-treated animals (n > 4). The numbers indicate the position of polyribosome peaks that are clearly detected in the control RNAi sample.

To further corroborate a potential role for either cytoPAP in the process of translation, we used sucrose gradient centrifugation to separate initiation from post-initiation ribonucleoprotein complexes and assessed a potential co-sedimentation of GLD-2 and GLD-4 with either fraction (Figure 6). To reveal the distribution of specific proteins across the gradient, we probed for cytoplasmic poly(A)-binding protein (PABPC), translation initiation factor 2α (eIF2α), both cytoPAPs and the GLD-4-specific cofactor, GLS-1. PABPC is part of initiation and post-initiation mRNA complexes while bound to the poly(A) tail (40). In *C. elegans*, two genes encode PABPC, *pab-1* and *pab-2* (41,42). eIF2α mediates the association of the initiator tRNA with the small ribosomal subunit and serves as a marker for translation initiation complexes (43). In *C. elegans*, eIF2α is encoded by *Y37E3.10* (23).

After gradient centrifugation, proteins from individual fractions of wild-type animals were isolated (Figure 6A). To monitor the efficiency of protein precipitation, we added a purified GST peptide that also served as a loading control. Consistent with PABPC as part of initiation and post-initiation complexes, both *C. elegans* PAB proteins were abundantly detected in light and heavy fractions (Figure 6C). By contrast, eIF2α (Y37E3.10), which is expected to function only during translation initiation, is more abundant in non-polysomal than polysomal regions of the gradient (Figure 6C). The cytoPAP GLD-2 is highly abundant in non-polysomal regions and only a small fraction of the total protein is detected in polysomal regions (Figure 6C). The cytoPAP GLD-4 is also abundantly present in non-polysomal regions but, interestingly, an additional accumulation of the protein is detected in polysomal regions (Figure 6C). A similar observation is made for its cofactor GLS-1 (Figure 6C). Together this suggests that GLD-4 and GLS-1, but less so GLD-2, might associate with post-initiation complexes.

To gain further support that the polysomal migration pattern of GLD-4 and GLS-1 depends on polysome formation, we analyzed protein distribution in EDTA-treated extracts (Figure 6B). Under these conditions ribosomes are disassembled into 40S and 60S subunits, and all signals in the heavy fractions for both PABs, GLD-4 and GLS-1 disappeared (Figure 6B and D). Although EDTA treatment may also affect mRNP assemblies other than polysomes, this result is consistent with a likely association of GLD-4 and GLS-1 with post-initiation complexes.

To test whether the differential enrichment of both cytoPAPs in polysomal fractions has any functional relevance on polysome formation, we compared general ribosome distribution profiles from staged wild-type, control RNAi, *gld-2(RNAi)*, *gld-4(RNAi)*, *gld-4(ef15)* and *gls-1(ef8)* animals. No significant difference was detected by comparing *gld-2(RNAi)* with control RNAi (Figure 6E). However, in *gld-4*-compromised animals, a clear decrease of the polysomal signal was detected (Figure 6F and Supplementary Figure S4A), and this defect was also observed in *gls-1(ef8)* mutants (Supplementary Figure S4B). Taken together, these data suggest that GLD-2 has a minor role in promoting general translation. By contrast, GLD-4 may actively promote general translational efficiency, most likely together with GLS-1.

## DISCUSSION

Cytoplasmic poly(A) polymerases represent an ancient class of nucleotidyltransferases that function as activators of post-transcriptional gene expression. We found that in *C. elegans*, the two cytoPAPs, GLD-2 and GLD-4, stimulate gene expression at the global level through distinct mechanisms. To a large part in germ cells, GLD-2 promotes the abundance of many translationally repressed mRNAs and facilitates bulk polyadenylation, whereas GLD-4 supports polysome formation. Our combined findings suggest that cytoPAPs globally employ diverse mechanisms to promote robust mRNA translation.

### GLD-2 is a bulk mRNA poly(A) polymerase that stabilizes translationally repressed mRNAs

Across species, GLD-2 polyadenylates specific mRNAs that encode genes important for germ cell development and neuronal function (10). Initially restricted to a handful of mRNAs targets (12,13,18,44–46), the list of potential GLD-2 targets was recently expanded by two microarray studies in worms and flies (14,18). As both studies used PAT assays to measure gene-specific poly(A) tail-length changes, global changes in poly(A) lengths remained unclear. Moreover, PCR-based PAT assays notoriously underestimate the true length of poly(A) tails and exact measurements are difficult (15). Therefore, we used a method that allows bulk poly(A) measurements free of any amplification bias (26). Our analysis documented a strongly reduced bulk poly(A) tail profile in *gld-2*-compromised animals. The most prevalent change was associated with primarily germline-derived mRNAs and revealed that GLD-2 is responsible for maintaining bulk poly(A) tail lengths beyond ∼35 nucleotides up to ∼90–100 adenosines. This magnitude of GLD-2 activity and a similar reduction to ∼40 nucleotides was also reported from other organisms. In *Drosophila*, the GLD-2 ortholog Wispy promotes tail extension of many mRNAs during late stages of oogenesis and early stages of embryogenesis (18). This argues that at least in germ cells GLD-2-type enzymes are major contributors to global cytoplasmic poly(A) metabolism.

Consistent with our bulk poly(A) measurements, our study also identifies a large set of GLD-2-stabilized germline mRNAs, and potentially expands a previous list of putative GLD-2 target mRNAs significantly. A previous study reported 538 GLD-2-associated mRNAs (14), of which 272 mRNAs are significantly stabilized in our study, representing an overlap of ∼50%. Yet, when focusing on germline-enriched RNAs (32), the number of GLD-2-associated mRNAs drops to 236 and the number of GLD-2-stabilized mRNAs among these drops further to 56, representing an overlap of ∼24%. However, we found that 444 germline-enriched mRNAs are less abundant in *gld-2(RNAi)*. Extrapolating from these numbers, we assume that the number of direct GLD-2 mRNA targets in the germ line may be much larger. Several possible explanations might account for this difference. First, the previous work was based on RIP-chip analysis (14). Therefore, it is possible that the co-immunoprecipitation procedure was not comprehensive. Furthermore, it is likely that our germline filter is too stringent and potential targets are consequently excluded from our analysis. Finally, although there is a strong positive correlation between GLD-2 expression and GLD-2 target mRNA abundance in our experiments, we cannot exclude that our extended list of GLD-2-regulated mRNAs might also contain mRNAs that are indirectly changing as a consequence of developmental changes, such as late spermatogenesis and oogenesis genes. Nonetheless, we propose that our list of GLD-2-stabilized germline genes is highly enriched for direct targets, which together with the previous data represents a more complete resource for studying GLD-2-regulated mRNAs.

GLD-2-mediated mRNA regulation is important for various aspects of germ cell development. Although not the focus of this study, we noticed that genes functioning early in prophase I are significantly reduced in *gld-2(RNAi)*. So far the only known gene in this category was *gld-1* (13). We suggest that mRNAs, such as *syp-1*, *him-3*, *htp-1* or *daz-1*, might also be GLD-2 targets. On a more global scale, an mRNA stabilizing function late in prophase I was proposed for a GLD-2 sub-complex (14). By analogy, we would like to extend this thought and propose that GLD-2, most likely as part of other sub-complexes, targets also a broad range of mRNAs during early stages of prophase I. This suggests that GLD-2 impacts mRNAs at all stages of germ cell development past meiotic entry.

The regulated balance between translational repression and activation supports germline organization and function across species. GLD-2-type cytoPAPs are broadly expressed during germ cell development and likely activate gene expression (7,45). In the adult *C. elegans* germline tissue, germ cells are organised in a strict spatial and temporal manner of gametogenesis, and several translational repressors promoting germ cell development are stage specifically expressed: the PUF-protein family member FBF-1 in pre-meiotic cells, the STAR protein family member GLD-1 in early meiotic prophase and the TIS11 zinc-finger protein family member OMA-1 in late meiotic prophase (47–49). By comparison, GLD-2 protein expression is much broader: it is low in pre-meiotic cells, steadily increases during early meiotic prophase and is most abundant in late meiotic prophase (7,19). Therefore, our finding that ∼77% of GLD-2-stabilized germline mRNAs are suggested targets of all three translational repressors (38,39,50) (C. Spike and D. Greenstein, personal communication) is intriguing. Given the low translational efficiency of GLD-2 targets, this observation further suggests that GLD-2 activity is primarily important for translationally repressed mRNAs. Hence, GLD-2's broad protein expression across the adult germline tissue paired with the strong mRNA target overlap of the local translational repressors indicates that GLD-2 might polyadenylate mRNAs during almost all stages of germ cell development. This argues for a central role of GLD-2-type cytoPAPs as global positive mRNA regulators that may oppose many translational repressors across species.

Beyond 3′end poly(A) extension to promote mRNA stability, the precise molecular mechanism and timing of GLD-2's enzymatic activity remains speculative. Many translational repressors are known to recruit poly(A) shortening enzymes (deadenylases) as part of their repressive function (51); prime examples are PUF proteins that associate with deadenylases from yeast to human cells (51). As deadenylation can initiate mRNA decay, GLD-2 might counteract this directed poly(A) shortening to promote mRNA stability during repression. In this scenario, a constant battle between deadenylation and polyadenylation could be envisioned, similar to the proposed antagonistic mechanism in *Xenopus* oocytes between the deadenylase PARN and xGld2 (44). Alternatively, GLD-2 activity may stabilize mRNAs after the switch from repression to activation, as short-tailed mRNAs released from repression are most likely prone to mRNA degradation. In either case, directed or globally occurring GLD-2-mediated polyadenylation is expected to protect mRNAs subject to translational control. In general, we suggest that GLD-2 represents a potent counterbalance to deadenylases employed by translational repressors.

Could GLD-2 have a role beyond mRNA stabilization? Long poly(A) tails are also important for translation efficiency (3,52). Throughout our global analysis, we find no major influence of *C. elegans* GLD-2 on the translatability of target mRNAs. However, the resolution power and the sensitivity of our analysis have to be taken into consideration here. In our genome-wide analysis, we split the gradient samples only into two fractions that allowed us to detect coarse redistributions from the polysomal to the non-polysomal regions, and vice versa. Certainly, we would have missed shifts occurring within the polysomal fractions, which would account for a more graded change of translational efficiencies, reflecting a high or lower translational initiation rate. Moreover, the majority of GLD-2 targets were low in abundance in the polysome region to begin with, arguing that the use of more sensitive techniques, such as higher resolution sucrose gradients paired with ribosome footprinting (36), might reveal a potential role of GLD-2 in stimulating mRNA translation. Hence, it remains possible that the primary effect of *gld-2* loss is translational repression combined with an enhanced secondary stimulation of mRNA degradation that overshadows positive translational regulation effects of GLD-2 function.

### GLD-4 promotes general translation

The non-canonical nucleotidyltransferase GLD-4 is evolutionarily most closely related to members of the conserved TRF4 family (10). In yeast, flies and mammalian cells, TRF4 homologous proteins represent the catalytic subunits of a nuclear RNA surveillance complex that adds short poly(A) tails of ∼10 nts to its RNA substrates (53). Moreover, the mammalian TRF4 paralog, hGld4/PAPD5, localizes to the cytoplasm where it forms a complex with the RNA-binding protein CPEB, promoting polyadenylation-induced translation of the tumor suppressor p53 (9). In *C. elegans*, the TRF4 ortholog GLD-4 predominantly localizes to the cytoplasm where it forms a protein complex with the nematode-specific protein GLS-1 to promote the translation of the germ cell-specific tumor suppressor GLD-1 and the Notch receptor GLP-1 (8,19). Despite these few examples, surprisingly little is known about additional mRNA targets and the global roles of cytoplasmic GLD-4-type nucleotidyltransferase in poly(A) tail metabolism and post-transcriptional mRNA regulation.

Consistent with gene-specific poly(A) tail measurements in *gld-4* mutants (15), we found in our bulk poly(A) tail measurements that many mRNAs in *gld-4*-compromised animals have slightly shorter tails. This observation is in sharp contrast to the strong polyadenylation defects of *gld-2*-deficient animals at the bulk and gene-specific level (15,16). This suggests that GLD-4 seems to have retained the enzymatic properties of the nuclear TRF4 proteins, adding rather short adenosine stretches to its targets. A subcellular relocation of GLD-4-type nucleotidyltransferases coupled to novel interactions with additional cytoplasmic factors might therefore represent alternative ways to regulate gene expression in evolution. It will be interesting to see whether the enzymatic activity of *C. elegans* GLD-4 is similarly weak among other cytoplasmic TRF4-type nucleotidyltransferases reported from other organisms (9). Although we cannot exclude that cytoplasmic GLD-4 promotes strong polyadenylation of specific targets, our bulk poly(A) tail measurements argue that GLD-4 contributes little to overall poly(A) tail metabolism. This suggests that GLD-2 and GLD-4 have different enzymatic activities *in vivo*, promoting strong or weak poly(A) addition, respectively, which may result from potential structural differences in their catalytic domains and distinct protein interactions to additional factors.

The functional consequences of GLD-4-mediated polyadenylation are less clear. Nuclear TRF4-mediated poly(A) addition is a prerequisite for RNA substrate degradation (54). However, in GLD-4-depleted animals, we detect no major changes in mRNA abundance, arguing that tail extension via GLD-4 cytoPAP does most likely not influence mRNA degradation. Instead, our overall data indicate that GLD-4 has a potential role in promoting translation. We find that GLD-4 cytoPAP and its co-factor GLS-1 is associated with putative translating ribosomes, and the loss of either protein leads to a strong reduction in polysome formation. Although it remains to be shown whether GLD-4-mediated mRNA polyadenylation is required for polysome assembly, it is attractive to speculate that GLD-4 could promote translation re-initiation by counteracting the proposed erosion of the poly(A) tail during active translation (55). Certainly, we find it less likely that a reduction of translation factor expression may indirectly affect polysome formation efficiency, as we did not find their mRNAs strongly reduced in *gld-4(RNAi)* animals. Alternatively, GLD-4 may aid the translation of mRNAs in a poly(A) polymerase-independent manner by a yet to be identified mechanism.

### Different cytoPAP mechanisms may represent a functional basis for robust and distinct gene expression control

The *C. elegans* germ line is a complex tissue that regulates its protein production primarily at the post-transcriptional level. Hence, efficient protein production is achieved via mRNA regulation. In order to satisfy the differential protein expression needs of germ cells during their developmental stages, we propose that a distinct utilization of GLD-2 and GLD-4 mechanisms would make it easy to combine or separate their activities in gene expression regulation. For example, different mRNAs could be more susceptible to one or the other mechanism. Alternatively, the combined GLD-2 and GLD-4 mechanisms might ensure that protein production is highly efficient, promoting the synthesis of large amounts of proteins in a short period of time. Such synergism is evident by the requirement of the two cytoPAPs to maintain high levels of GLD-1 protein in the germ line to ensure meiotic commitment (8). Furthermore, the overlap between *gld-2(RNAi)*-decreased and *gld-4(RNAi)*-less translated genes suggests that the combined activity of both cytoPAPs might be important for a specific set of mRNAs. In order to get a deeper understanding of the relationship between the GLD-2 and GLD-4, more work is needed to reveal their precise mechanisms. In general, we suggest that the diversification of cytoPAP mechanisms represents an additional regulatory asset to all biological systems that utilize post-transcriptional gene regulation.

## ACCESSION NUMBERS

NGS data have been deposited in the GEO database (accession number: GSE58918).

## SUPPLEMENTARY DATA

Supplementary Data are available at NAR Online.

SUPPLEMENTARY DATA
