# Supplementary material for: The cytoplasmic poly(A) polymerases GLD-2 and GLD-4 promote general gene expression via distinct mechanisms
Source: Nucleic Acids Res. 2014 Sep 12;42(18):11622–33. doi: 10.1093/nar/gku838 (PMC4191412; doi:10.1093/nar/gku838)
Supplement: SUPPLEMENTARY DATA [file supp_42_18_11622__index.html]

The cytoplasmic poly(A) polymerases GLD-2 and GLD-4 promote general gene expression via distinct mechanisms — The cytoplasmic poly(A) polymerases GLD-2 and GLD-4 promote general gene expression via distinct mechanisms — SUPPLEMENTARY DATA 

# The cytoplasmic poly(A) polymerases GLD-2 and GLD-4 promote general gene expression via distinct mechanisms

## SUPPLEMENTARY DATA

**Files in this Data Supplement:**

- SUPPLEMENTARY DATA
- SUPPLEMENTARY DATA
