# Supplementary material for: The cytoplasmic poly(A) polymerases GLD-2 and GLD-4 promote general gene expression via distinct mechanisms
Source: Nucleic Acids Res. 2014 Sep 12;42(18):11622–33. doi: 10.1093/nar/gku838 (PMC4191412; doi:10.1093/nar/gku838)
Supplement: SUPPLEMENTARY DATA [file supp_gku838_nar-01698-v-2014-File008.pdf]

The cytoplasmic poly(A) polymerases GLD-2 and GLD-4 promote general gene expression via distinct mechanisms

Marco Nousch, Assa Yeroslaviz, Bianca Habermann, and Christian R. Eckmann

Supplemental information

Figure S1. Global mRNA abundance changes in *gld-2(RNAi)* and *gld-4(RNAi)*.

Figure S2. Detection of bulk poly(A) tail changes in the germ line.

Figure S3. Overlap of putative *gld-2*– and *gld-4*–sensitive mRNA targets.

Figure S4. *gld-4* and *gls-1* promote polysome formation.

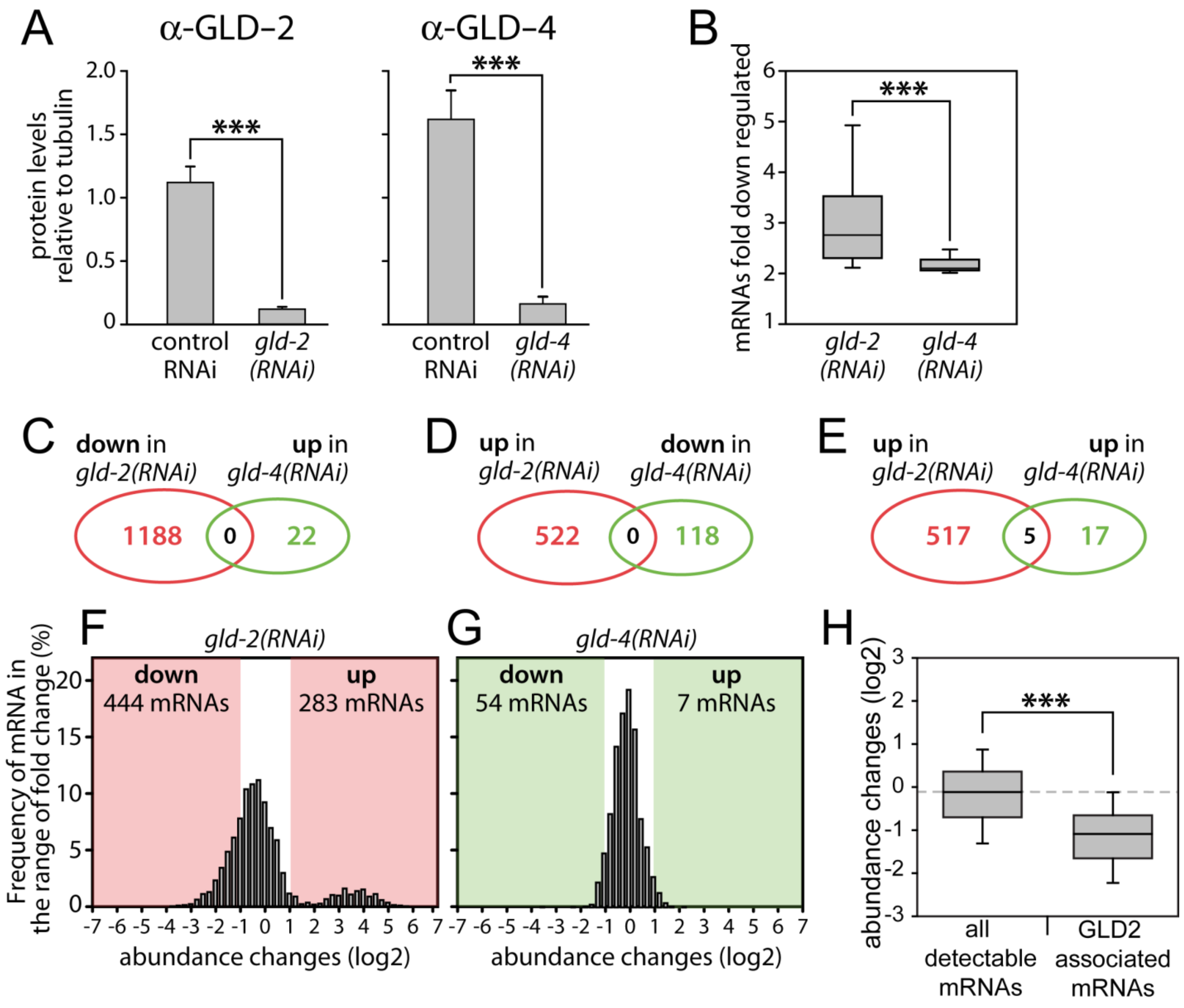

Figure S1. Global mRNA abundance changes in *gld-2(RNAi)* and *gld-4(RNAi)*. (A, B and I) Student's t-test: ***, p<0.001; n.s., not significant. (**A**) Measurements of the RNAi knockdown efficiency as quantified by Western blotting (n=3). (**B**) mRNA abundance changes in *gld-2(RNAi)* and *gld-4(RNAi)* of significantly down regulated mRNAs. Shown is the fold change of 95 percentile of the data. The median is indicated. (**C** – **E**) The overlap of mRNAs that significantly change in their abundance in *gld-2(RNAi)* and *gld-4(RNAi)* are shown. (**F** and **G**) Abundance changes of germline-enriched mRNAs (32) in (F) *gld-2(RNAi)* or (G) *gld-4(RNAi)*. (**H**) Abundance changes of all detectable mRNAs shown in Fig. 1E and GLD-2 associated mRNAs shown in Fig. 2A. Shown is the log2 of the fold change of 95 percentile of the data sets.

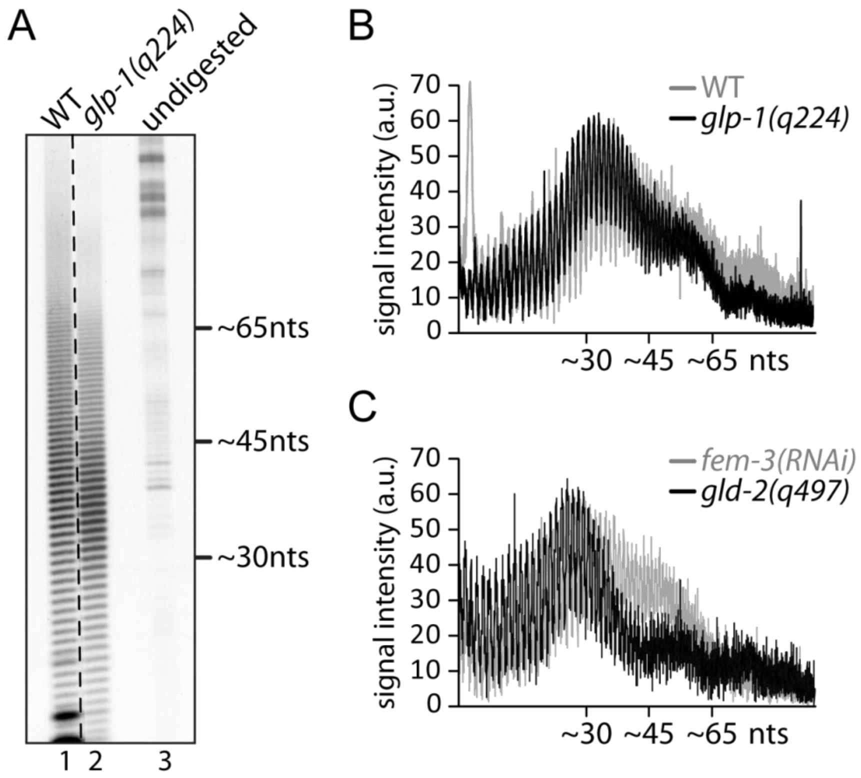

Figure S2. Detection of bulk poly(A) tail changes in the germ line. (**A** and **B**) Measurements of wild-type (WT) and germline-less *glp-1(q224ts)* animals grown at 25°C from the L1 to the young adult stage. (**A**) Polyacrylamide gel of bulk poly(A) tails. Lane 3 is a sample of labeled wild-type RNA prior to RNAse A/T1 digestion. (**B**) Line scan of lanes 1 and 2 in (A). (C) Line scan of signal distribution of *fem-3(RNAi)* and *gld-2(RNAi)* shown in Fig. 2*B*.

A

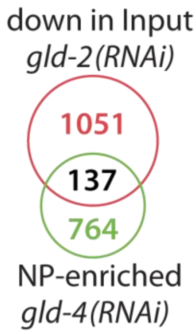

B

overlap	GO terms		p-value
gld-2(RNAi) Input & *gld-4(RNAi)* NP enriched	GO:0031497	nucleosome organisation	8.6 E-4
	GO:0009792	embryonic development ending in birth or egg hatching	5.1 E-3
	GO:0007049	cell cycle	5.9 E-3

Figure S3. Overlap of putative *gld-2*– and *gld-4*–sensitive mRNA targets. (**A**) The overlap between less abundant mRNAs in *gld-2(RNAi)* and mRNAs that shift towards the NP-fraction in *gld-4(RNAi)* is shown. (**B**) GO-term analysis of the overlap shown in (A).

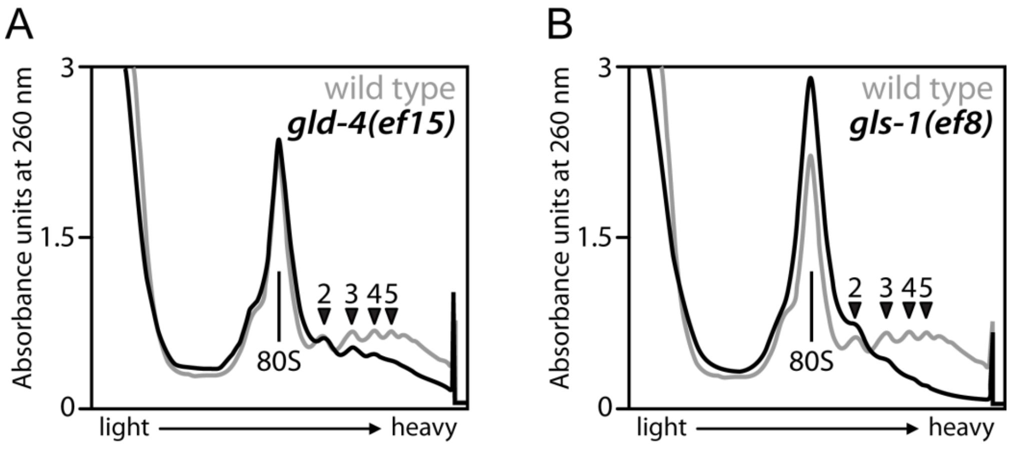

Figure S4. *gld-4* and *gls-1* promote polysome formation. (**A**) Absorbance profile of wild-type (WT) and *gld-4(ef15)* mutant animals. (**B**) Absorbance profile of *gls-1(ef8)*. Profiles were recorded from at least two independent experiments.
